# Association between surgery with anesthesia and cognitive decline in older adults: Analysis using shared parameter models for informative dropout

**DOI:** 10.1017/cts.2020.519

**Published:** 2020-08-04

**Authors:** Katrina L. Devick, Juraj Sprung, Michelle Mielke, Ronald C. Petersen, Phillip J. Schulte

**Affiliations:** 1 Department of Health Sciences Research, Division of Biomedical Statistics and Informatics, Mayo Clinic College of Medicine and Science, Scottsdale, AZ, USA; 2 Department of Anesthesiology and Perioperative Medicine, Mayo Clinic College of Medicine and Science, Rochester, MN, USA; 3 Department of Health Sciences Research, Division of Epidemiology, Mayo Clinic College of Medicine and Science, Rochester, MN, USA; 4 Department of Neurology, Mayo Clinic College of Medicine and Science, Rochester, MN, USA; 5 Department of Health Sciences Research, Division of Biomedical Statistics and Informatics, Mayo Clinic College of Medicine and Science, Rochester, MN, USA

**Keywords:** Missing not at random (MNAR), shared parameter models, informative dropout, general anesthesia, surgery, older adults, cognitive *z*-scores

## Abstract

**Objectives/Goals:**

The association between surgery with general anesthesia (exposure) and cognition (outcome) among older adults has been studied with mixed conclusions. We revisited a recent analysis to provide missing data education and discuss implications of biostatistical methodology for informative dropout following dementia diagnosis.

**Methods/study population:**

We used data from the Mayo Clinic Study of Aging, a longitudinal study of prevalence, incidence, and risk factors for mild cognitive impairment (MCI) and dementia. We fit linear mixed effects models (LMMs) to assess the association between anesthesia exposure and subsequent trajectories of cognitive *z*-scores assuming data missing at random, hypothesizing that exposure is associated with greater decline in cognitive function. Additionally, we used shared parameter models for informative dropout assuming data missing not at random.

**Results:**

A total of 1948 non-demented participants were included. Median age was 79 years, 49% were female, and 16% had MCI at enrollment. Among median follow-up of 4 study visits over 6.6 years, 172 subjects developed dementia, 270 died, and 594 participants underwent anesthesia. In LMMs, exposure to anesthesia was associated with decline in cognitive function over time (change in annual cognitive *z*-score slope = −0.063, 95% CI: (−0.080, −0.046), *p* < 0.001). Accounting for informative dropout using shared parameter models, exposure was associated with greater cognitive decline (change in annual slope = −0.081, 95% CI: (−0.137, −0.026), *p* = 0.004).

**Discussion:**

We revisited prior work by our group with a focus on informative dropout. Although the conclusions are similar, we demonstrated the potential impact of novel biostatistics methodology in longitudinal clinical research.

## Introduction

Recent research studies have reported that surgery with general anesthesia is associated with subsequent cognitive decline in older adults [1,2]. However, several studies have reported contradictory results [3,4]. Further supporting the hypothesis that anesthesia is associated with subsequent cognitive decline is a preclinical study in which cell lines exposed to inhalational anesthesia demonstrate enhancement of brain neuropathology implicated in Alzheimer’s dementia [5] and a systematic review of short-term perioperative cognitive dysfunction [6]. Recent clinical research studies have used longitudinally collected data to assess this hypothesis. Longitudinal studies obtain repeated assessments on subjects over time. For example, longitudinal data could be collected at several scheduled study visits or at multiple encounters with the medical system. While a sample of subjects may be enrolled into a longitudinal study either prospectively or by defining a retrospective cohort, some subjects may drop out, as the study progresses during the follow-up period.

Subjects with severe cognitive impairment or dementia more frequently drop out from longitudinal research studies [7,8]. This may result from elevated caregiver burden for persons with cognitive impairment or dementia [9,10]. Furthermore, participants with dementia are also less able or even unable to complete a lengthy neuropsychological testing battery. As a result, subjects with poorer cognitive outcomes may be more likely to be missing their outcome measurements in the research study. This type of dropout from a longitudinal study is *informative*, if the missing values are related to unobserved data. If informative dropout is present, associations estimated from statistical analyses that do not account for missing not at random data are biased.

This manuscript revisits the recent study by Schulte *et al.* (2018), assessing the association between surgery or procedures requiring general anesthesia and subsequent cognitive decline in older adults [11]. In their paper, an ad-hoc approach to missing data and possible informative dropout was taken. They performed a sensitivity analysis restricted to the first 4 years of follow-up before substantial dropout was observed. However, with recent information about the potential subject dropout process in the Mayo Clinic Study of Aging (MCSA), we decided to reanalyze these data from the same data sources using statistical methodology that allows for informative dropout. The aim of this manuscript is to demonstrate the impact of biostatistics methodology decisions on clinical research outcomes and expound on biostatistics methods for missing data available to clinical researchers in the longitudinal data setting. Specifically, we address implications of dropout in longitudinal studies and implement shared parameter models to reassess the association between surgery with general anesthesia and cognitive function in the presence of informative dropout.

### Missing Data in Clinical Research

In any study where missing data are present, the underlying reason or mechanism for why these data are missing is important to consider. Estimates using partially observed data are usually less efficient than those using fully observed data, where efficiency is defined by the precision or variability of an estimate. In addition, missing data may cause bias. Bias and variance have been described previously using the analogy of a dartboard. When darts are thrown, they may be narrowly clustered near each other in tight formation (low variability) or spread out (high variability). Furthermore, they may be centered around the bullseye (unbiased) or may have a center toward an edge (biased). In select situations, observations with missing data can be ignored, and statistical analyses based only on the observed complete-case data will provide an unbiased estimate, even if less efficient than what could have been obtained with fully observed data (no missingness). However, this is rare in practice, and statistical methods may need to consider the underlying reasons for missing data. In the following description of missing data types and methods, we focus on the issue of bias.

In 1976, Rubin distinguished between ignorable and nonignorable mechanisms and defined three types of missing data: missing completely at random (MCAR), missing at random (MAR), and missing not at random (MNAR) [12]. Data are MCAR if the subset of data that is complete consists of random observations from the whole dataset. In other words, the missing data have no relationship with any data in the dataset, observed or missing. An example of MCAR data may include a person who is lost to follow-up due to relocation or a study where only a random subset of patients are selected for expensive genotyping. In the setting of MCAR, analyses performed only on the complete data are valid, meaning that if all other statistical assumptions are satisfied, the model will provide unbiased estimates.

When data are MAR, the missing data depend on the observed data but do not depend on unmeasured factors or missing data. An example of MAR is if subjects receiving a novel therapy stop attending follow-up study visits more often than those receiving standard care, so that missing data at later visits are related to the observed treatment group. Analyses that incorporate all observed information, and not only people with complete data, may provide valid estimates. Common approaches to MAR data include weighting using an estimated probability of missingness and multiple imputation. MAR and MCAR are sometimes called *ignorable* missing data, since statistical modeling or assumptions about the missing data mechanism, or underlying reason for missingness, are not required.

When missing values depend not only on the observed data, but also on the unobserved data, the missing data mechanism is considered MNAR. This typically arises when the missing data point is missing directly because of the unobserved value. Self-reported income is a common example, where study participants at the extremes may be self-conscious about reporting their income. In the absence of other available data about socioeconomic status, missing income would be directly dependent on the unobserved and unreported income values. In our analysis, a concern is whether the data we are missing after subjects drop out, or stop attending or completing study visits, represent unobserved lower cognitive scores. The reasoning, based on discussions with subject experts and MCSA investigators, is that subjects with lower cognitive function require additional care, and attending research appointments may represent a burden. MNAR data are considered *nonignorable*, and methods for analyzing MNAR data require statistical modeling or assumptions about the missing data process. Of note, while a dataset can be used to determine whether data are MAR versus MCAR, it is not possible to differentiate between MAR and MNAR using observed data. Instead, this information may come from auxiliary sources, such as research staff who may have a better understanding of why data were not obtained or why subjects did not attend appointments.

Missing data are a prevalent problem in longitudinal studies, since repeated measurements are collected on the same individuals over time requiring multiple contacts with the subjects. As participants are followed over time, there are more opportunities for information to be missing. People can drop out of a study and have missing data for the remainder of the study, or they could miss one scheduled visit and have complete data for the rest of the study or some combination of these occurrences. There is extensive literature on methods for missing data in a longitudinal setting [13–22].

In our motivating data analysis, we are concerned that subjects who drop out have different longitudinal trajectories of the outcome compared to subjects who do not drop out, and those differences cannot be explained by other observed data. There are three common approaches for informative dropout data: pattern-mixture, selection, and shared parameter models [23]. There are many resources extensively describing methodology for pattern-mixture models [23–27], selection models [20,23,28], and shared parameter models [29–32] broadly. All three approaches have been used in practice to account for informative dropout in aging populations, in many cases assuming informative dropout related to dementia [33–38]. A limitation of pattern-mixture and selection models is that time is typically discrete. Thus, they are useful when the timing of study visits is consistent for all subjects (e.g., first visit at 6 months, second visit at 12 months, third visit at 18 months, etc., so that visits could be binned by visit number). If subjects are not observed at the same time intervals, and thus the time scale is continuous, then pattern-mixture and selection models are not straightforward to implement with available software. Shared parameter models, however, allow for time on a continuous scale. Since dropout times in our motivating example are continuous, the focus of this paper will be on implementing shared parameter models to account for informative dropout.

A shared parameter approach jointly models the longitudinal outcome and time-to-dropout simultaneously. It gets its name from the sharing of information between the two models while estimating the model parameters. Specifically, some parameters are found in both models. For example, one can jointly fit a linear mixed effects model to the longitudinal outcome and a survival model, such as Cox proportional hazards regression, for time-to-dropout. In the shared parameter approach, the survival model may include the random effects or time-dependent estimates from the longitudinal outcome model. Linear mixed effects models fit via maximum likelihood allow for data to be MAR while still providing valid inference (unbiased estimates), assuming other assumptions hold such as no unmeasured confounders. Shared parameter models allow for data to be MNAR by updating the subject specific random intercepts and/or slopes in the linear mixed effects model by jointly modeling the longitudinal outcome with the time-to-dropout process. Intuitively, the shared parameter model revises the estimates in the linear mixed effects model accounting for the dropout process. In our setting, we are interested in modeling trajectories of longitudinally collected cognitive scores as a function of anesthesia exposure while considering possibly informative dropout due to dementia or death. The shared parameter model we consider allows for MNAR cognitive scores by jointly modeling cognitive score trajectories and time-to-dementia or death.

## Materials and Methods

### Data

Study subjects were enrolled in the Mayo Clinic Study of Aging (MCSA), a prospective epidemiologic longitudinal cohort study of the prevalence, incidence, and risk factors for mild cognitive impairment (MCI) and dementia among Olmsted County, Minnesota residents [39]. We included MCSA participants who were at least 70 years of age and without dementia at enrollment. We used enrollment dates of this cohort from October 1, 2004 to December 31, 2009. Recent enrollment cohorts continue to expand the MCSA sample and are not included in the current study. Follow-up MCSA visits were included through December 31, 2014.

Details of the MCSA study have been described elsewhere [39]. In brief, subjects had a clinical evaluation at an enrollment visit, and follow-up visits were scheduled at approximately 15-month intervals thereafter. While planned follow-up was at 15 months, actual visits occurred more irregularly. Follow-up visits typically occurred 12–18 months after the prior visit; however, some subject’s visits were outside of this interval. As a result, investigators hesitate to bin follow-up visits into discrete time points or visit numbers. Apolipoprotein ϵ4 (*APOE* ϵ4) allele (apolipoprotein E genotype; *APOE*) genotyping was performed at enrollment. Each study visit included an evaluation by a study coordinator or nurse consisting of medical history, memory by self-report, and family history of dementia. A neurological assessment was performed at each visit by a physician. In addition, a psychometrist administered a cognitive testing battery of nine tests covering four cognitive domains at each visit. For our purposes, we define a global cognitive *z*-score summarizing overall cognitive function across the four domains, as described previously [11,39,40]. Briefly, a subject’s score represents the number of standard deviations their score is from the mean score of a reference population of cognitively unimpaired subjects of an MCSA 2004 enrollment cohort. After each clinic visit, a consensus conference reviewing all study information was conferred. The consensus conference included the study coordinator or nurse, physician, and neuropsychologist involved with the participant’s visit as well as neurologists and other study staffs [39,41,42].

MCSA data were combined with surgery and procedure data performed under general anesthesia retrospectively identified through the Rochester Epidemiology Project (REP) [43]. Procedures with anesthesia through 2014 were further manually reviewed, and the data abstracted were combined with MCSA visit data [44]. Mortality data were also obtained from the REP.

### Statistical Analysis

Missing data for baseline subject covariates were rare in the MCSA data, with nine subjects (0.5% of the eligible sample) excluded due to missing alcohol status (*n* = 4) or missing APOE ϵ4 status (*n* = 5). We assumed these few values were MCAR in the current study to focus on the issue of potential informative dropout. Median follow-up (and quantiles) was estimated using the reverse Kaplan–Meier method.

Our primary hypothesis was that exposure to surgery with general anesthesia is associated with worse trajectory of cognitive *z*-scores over time. The association between surgery with general anesthesia and longitudinally measured global cognitive *z*-scores was first analyzed using linear mixed effects models (LMM) under the assumption that dropout is MAR. Fixed effects in the LMM included baseline covariates, time in years since enrollment, the interaction between baseline covariates and time since enrollment, and time after a post-enrollment surgery with general anesthesia. Time in years since enrollment was modeled to have a linear relationship with cognitive *z*-scores, since in prior studies, we found that a linear relationship described the data well. Time since enrollment, and its interactions with baseline covariates, estimated the average annual rate of change in cognitive *z*-scores without or prior to surgery with general anesthesia. Time after a post-enrollment surgery with general anesthesia was the primary exposure variable to test the hypothesis that exposure is associated with a change in the slope of cognitive *z*-scores over time. Time after a post-enrollment surgery with general anesthesia was a time-dependent variable that was zero among all subjects without or prior to surgery with general anesthesia and begins counting time, in years, after surgery with general anesthesia. Thus, the coefficient for time after post-enrollment surgery with general anesthesia estimated the change in the average annual slope of cognitive *z*-scores following a subject’s post-enrollment surgery. In all analyses, we adjusted for potential confounders identified *a priori* by investigators including: age at enrollment, sex, education level, APOE ϵ4 status, midlife diabetes mellitus, midlife hypertension, midlife dyslipidemia, atrial fibrillation, Charlson Co-morbidity Index, history of congestive heart failure, stroke, coronary artery disease, marital status, smoking status, diagnosed alcohol problem, and baseline MCI status. We also adjusted for exposures to surgery with general anesthesia in the 20 years prior to enrollment. Interactions between time after enrollment and baseline covariates were further included. Random effects in the LMM included random intercepts, random slope, and random slope after anesthesia exposure. Other random effects structures were considered for the LMM, and we chose the model with best fit using the Akaike Information Criterion (AIC). In this initial model, we assumed that data were MAR conditional on the observed data including *z*-scores prior to dropout, surgery with general anesthesia, and adjustment variables. This analysis differs from Schulte *et al.* (2018), which assessed the relationship between post-enrollment exposure and cognitive outcomes with an LMM among those without exposure to anesthesia and surgery in the 20 years before enrollment by defining the time-dependent exposure only for those without prior anesthesia exposure [11]. Here, we estimated the association between exposure to anesthesia and surgery with subsequent cognitive trajectories with an LMM among all subjects.

Second, we used a shared parameter model, as described in the missing data section, to assess the association under the assumption of informative dropout. The shared parameter model combined two submodels. In the first step, we fitted an LMM submodel for the outcome of global cognitive *z*-score. The model was similar to the one specified above and used the same covariates, but for computation reasons, we excluded observations from subjects occurring after a dementia diagnosis, so that dementia always prompts dropout. In the next step, we fit a survival submodel for time to drop out due to death or dementia. Dropout due to death was defined by death within 18 months of a prior study visit. Subjects without dementia diagnosis or death were censored at last known alive or at the end of available follow-up (December 31, 2014). Variables in this survival submodel included the same adjustment variables at enrollment as in the LMM specified previously.

For the shared parameter model, we combined these two submodels by sharing information between the two models. Several options exist for the association structure of the two submodels. We chose an approach where each random effect term included in the LMM was also fitted in the survival model and, thus, explains the interdependencies between the models for the dropout process and the longitudinal trajectory of cognitive scores. Specifically, random intercept, slope, and change in slope after exposure parameters from the longitudinal model were covariates in the model for time to death or dementia. Alternative approaches were considered, and we used the deviance information criterion (DIC) to select the one shared parameter model that fit the data best. We used a Bayesian approach implemented in R statistical software and the *JMbayes* package (Joint Models Using Markov chain Monte Carlo in R) [45]. Other software options for shared parameter models exist in R [46], SAS [47,48], and Stata [49], among others. Markov chain Monte Carlo algorithms estimated the joint model by re-fitting both models under this new shared parameterization. The prior distributions for parameters used were the software package defaults – typical diffuse and non-informative priors [45].

In our approach, we allowed for dropout to be MNAR by modeling the dropout process related to death or dementia. Participants may have dropped out for other reasons, such as relocation. While studies with discrete follow-up visits could further include “other” dropouts, in the continuous time setting, we were not able to determine a date of dropout reliably. Hence, we assumed that the dropout for other reasons beyond death or dementia was MAR or MCAR, which were adequately handled in the current analysis approaches.

Results of our model were described, in part, with a figure displaying the estimated mean trajectory of four hypothetical subjects. Hypothetical subjects were chosen to illustrate variation in cognitive scores at enrollment and changes in cognitive scores over time. Mean trajectories for these four subjects used estimated coefficients from the shared parameter model, with random effects set to zero. Trajectories were estimated for the scenario where the subject had no post-enrollment exposure to surgery and general anesthesia and for the scenario where the subject had a surgery with general anesthesia at 2 years post-enrollment.

The current study used the same data source as Schulte *et al.* (2018), but we note the following differences between the current analysis and the previously published Schulte *et al.* (2018) analysis [11]. (1) We retained subjects with a single MCSA visit with cognitive score in the present analysis, whereas Schulte *et al.* (2018) inclusion criteria required two visits with cognitive scores. (2) We assessed whether there was an association between exposure to surgery with general anesthesia and subsequent cognitive decline among all patients in the cohort, whereas Schulte *et al.* (2018) assessed the association among those that are anesthesia-naïve in the 20 years before MCSA enrollment. Thus, our inference includes a broader population. (3) Cognitive scores observed after dementia were excluded in some analyses, as mentioned previously. Finally, (4) Schulte *et al.* (2018) used LMMs under the MAR assumption, whereas in the current analysis, we repeated the LMMs under MAR accommodating changes to (1)–(3) above and further analyzed the association of interest using shared parameter models assuming MNAR.

Additional details for the statistical model, including R programming code, can be found in the Supplemental Material.

## Results

Of 1948 non-demented MCSA participants, median [25th, 75th percentiles] age was 79 [74, 83] years, and 49% of subjects were female. Midlife hypertension and dyslipidemia were common (36% and 43%, respectively). Mild cognitive impairment was present in 16% at enrollment, and 27% of subjects were APOE ϵ4 positive. Participants had a median of 4 [3, 6] MCSA visits with cognitive scores (total 8417 observations among 1948 subjects) ascertained over 6.6 [4.2, 8.1] years in the study. Time between consecutive study visits was a median 1.3 years (approximately 15–16 months), but varied substantially (1st percentile = 0.5 years, 99th percentile = 2.7 years), and 25% of observations were outside ±2 months of the planned 15-month interval.

Development of dementia was observed in 172 subjects during the study period. Death within 18 months of a study visit was considered death on study before dropout for other reasons; 270 subjects died during follow-up in this study. Patient characteristics are described according to study outcomes in the Supplemental Material. In brief, those who died on study were older and had more comorbidities. Those who developed dementia more often had *APOE ϵ4* allele and mild cognitive impairment at enrollment. A total of 594 participants had post-enrollment exposure to surgery with general anesthesia during the study period. Characteristics of procedures have been described previously with significant overlap with the current study sample [11,50].

**Fig. 1:**
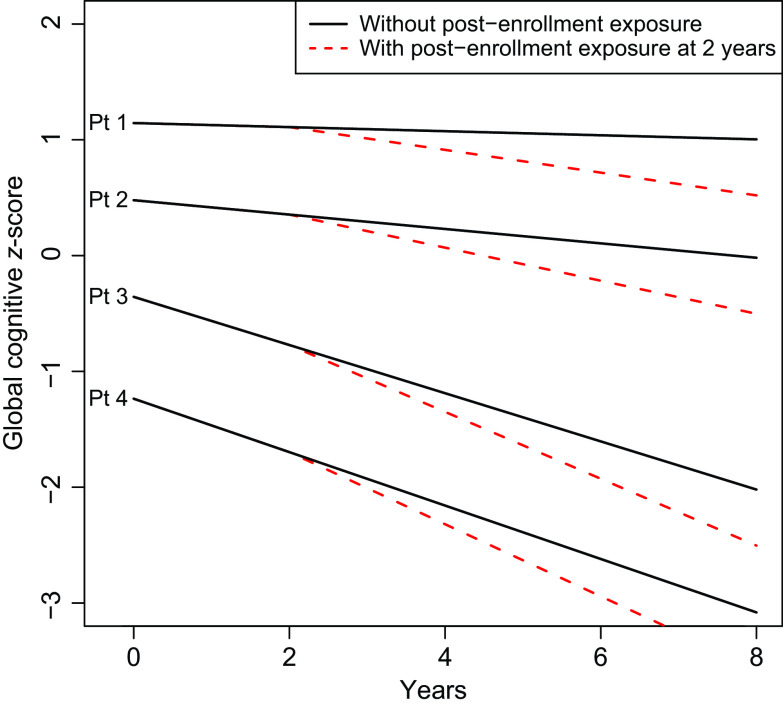
Simulated trajectories for four hypothetical patients under two scenarios: (1) no surgery with general anesthesia during the follow-up period (shown with solid line), and post-enrollment surgery and anesthesia at 2 years after enrollment (shown with dashed line). Follow-up is described from enrollment through 8 years. Exposure refers to exposure to surgery and general anesthesia. The four hypothetical patients were chosen to represent varying degrees of health at enrollment. Patient (Pt) 1 is a 75-year-old female, never a smoker, married, with ≥16 years of education, with Charlson comorbidity index of 1, APOE ϵ4 negative, cognitively normal at enrollment, and with prior exposure to anesthesia in the last 20 years. Pt 2 is an 80-year-old female, never a smoker, married, 13–15 years of education, with prior history of coronary artery disease and a Charlson comorbidity Index score of 2, APOE ϵ4 negative, cognitively normal at enrollment, and without prior exposure to anesthesia in the 20 years before enrollment. Pt 3 is an 85-year-old female, former smoker, single-partner status, 12 years of education, with prior history of stroke and atrial fibrillation and a Charlson comorbidity Index score of 3, APOE ϵ4 positive, cognitively normal at enrollment, and with a prior exposure to anesthesia in the last 20 years. Pt 4 is a 75-year-old male, current smoker, single-partner status, 12 years of education, prior history of coronary artery disease and a Charlson comorbidity Index score of 4, with midlife dyslipidaemia, APOE ϵ4 positive, mild cognitive impairment at enrollment, and without exposure to anesthesia in the 20 years prior. The plot demonstrates that changes over time attributable to surgery and anesthesia before enrollment or post-enrollment represent a subtle, although statistically significant, change in the average trajectory of cognitive *z*-scores relative to the variability in *z*-scores inherent in the population.

In linear mixed effects models under the assumption that dropout is ignorable (MAR), there was significant evidence to suggest that exposure to surgery and general anesthesia is associated with greater cognitive decline after surgery (change in annual slope of cognitive *z*-score = −0.063, 95% Confidence Interval (CI): (−0.080, −0.046), *p* < 0.001; Table 1). There were 63 MCSA visits with cognitive scores after a dementia diagnosis. When the analysis was repeated without these data and still assuming MAR, results were nearly identical (see Table 1). However, when refitted in the shared parameter approach for ignorable missing data (allowing for MNAR), estimates of the association with cognitive decline were more pronounced (change in annual slope = −0.081, 95% Credible Interval (CrI): (−0.137, −0.026), *p* = 0.004). These results suggest, on average, exposure to surgery with general anesthesia is associated with more than double the expected rate of change in cognitive decline compared to pre-exposure among older adults (see Table 1). The full model fit is described in the Supplemental Material. Since data were complete at enrollment, intercept-related terms that describe the relationship between covariates and the enrollment cognitive *z*-score were similar between models; however, slope-related terms that describe how covariates were associated with change in cognitive *z*-score over time tend to be larger (further from 0, the null hypothesis) in the shared parameter model, possibly reflecting *informative dropout*. Fig. 1 demonstrates simulated paths of four hypothetical subjects with varying risks for cognitive decline. As in prior studies by our team [11], the estimated changes in slope associated with surgery and general anesthesia are small, relatively, compared to variation in the population.

**Table 1.** Cognitive scores over time following enrollment

		Surgery and general anesthesia	
Method	Slope est.*	Difference in slope est. (95% CI)**	*p*
Linear mixed effects model	−0.072	−0.063 (−0.080, −0.046)	<0.001
Linear mixed effects model, excluding observations after dementia	−0.076	−0.063 (−0.079, −0.047)	<0.001
Shared parameter model§ – death or dementia	−0.077	−0.081 (−0.137, −0.026)	0.004
Shared parameter model§ – dementia alone	−0.065	−0.074 (−0.129, −0.019)	0.006

Linear mixed effects models assume data are missing at random (MAR), whereas shared parameter models assume missing not at random (MNAR).

*Slope est. is the estimated change in cognitive *z*-score slope per year without a post-enrollment exposure to surgery and anesthesia. It is obtained by averaging estimated subject-specific slopes over the distribution of the sample and assuming no post-enrollment exposure.

**Difference in slope est. is the average change in annual slope following a post-enrollment exposure to surgery with general anesthesia. The linear mixed effects model provides an estimate with 95% confidence interval. Shared parameter models provide an estimate and 95% credible interval.

§Shared parameter models exclude observations after dementia.

We decided, based on review of other papers implementing models for informative dropout, to focus on the composite dropout due to death or dementia. As an alternative, we further considered a shared parameter model where the survival model was fitted for the endpoint of dropout due to dementia alone. Thus, this analysis assumes dropout due to dementia is MNAR, but dropout due to death is MAR. Results were similar, but of smaller magnitude for the estimate (change in annual slope = −0.074, 95% CrI: (−0.129, −0.019), *p* = 0.006; Table 1).

## Discussion

We showed that when accounting for potential informative dropout due to death or dementia, the estimated association (point estimate) was 29% larger than the results under a MAR assumption, although both lead to similar qualitative conclusions. Results from all models we considered suggested that exposure to anesthesia and surgery is associated with greater decline in cognitive *z*-scores compared to those not exposed. When participants drop out from research studies after dementia diagnosis or death, missing data may represent informative missingness, and there may be some sensitivity for the association with respect to this type of missing data mechanism. Specifically, in our study, poorer cognitive outcomes may be unobserved or missing, and analyses using only observed outcome data prior to dropout may underestimate the true association.

Our results were qualitatively similar to Schulte *et al.* (2018) and Sprung *et al.* (2019) [11,50], which suggested an acceleration of cognitive decline beyond that expected for normal aging in this population of older adults. Under the linear mixed effects models in this study, our results suggested a point estimate that is stronger in the association between surgery with general anesthesia and cognitive outcomes compared to Schulte *et al.* (2018), but this may be attributable to the small differences in inclusion criteria and that our estimates include exposure among those with surgeries and general anesthesia prior to MCSA enrollment. Our shared parameter model, while estimating a slightly more pronounced association, had similar qualitative conclusions to these prior studies, despite new information about potential dropout processes in the MCSA. As a result, we refer the reader to those papers and references therein for discussion of the clinical implications of these results.

In this study, we implemented shared parameter models to model informative dropout. Other studies have taken different approaches, including pattern-mixture models [35,36,38]. In those studies, data had discrete observation times amenable to the pattern-mixture analysis approach. When follow-up is discrete, researchers could consider this approach. However, pattern-mixture models and selection models are not easily extended to continuous observation times. We did not consider pattern-mixture and selection models for our data, since an ad-hoc approach to binning data with ±2 months would result in deleting 25% of the longitudinal observations.

Our baseline data were nearly complete at enrollment due to MCSA procedures that comprehensively collect data on participants. However, nine subjects were excluded from our analyses for missing baseline data. When missing data are present, investigators need to consider potential causes and mechanisms for that missingness. In our study, we felt it reasonable to perform complete case analysis with respect to baseline covariates assuming MCAR to exclude less than half of one percent of the sample with missing baseline covariates, especially as it relates to missing APOE ϵ4 status as refusal to participate in genotyping is unlikely to be related to APOE ϵ4 status nor other covariates. However, other studies may need to consider additional methodologies for missing baseline covariates or missing exposure status.

Additional limitations apply to our study. First, the MCSA is a prospective cohort of older adults residing in Olmsted County, Minnesota, and results may not generalize broadly to other diverse populations. Furthermore, the MCSA was not designed for the specific investigation of exposures to anesthesia and surgery, and so the timing of MCSA assessments does not correspond to a clear time point relative to surgery with general anesthesia among subjects with exposure. In the current study, we fit models for time to death or dementia to describe dropout. Alternative approaches could define dropout due to other causes and alternative approaches exist for death as informative censoring [18,21,22]. This may be more feasible in the discrete visit time scenario. Additional approaches not considered here could further model the dropout process using competing risks models or multistate models that evaluate time to each potential dropout mechanism. We used a complete case analysis with respect to missing baseline data (excluding 0.5% of the sample) to maintain focus on the issue of informative dropout. However, other studies with more significant missing baseline data may want to consider alternative approaches for those missing data. Residual confounding may be present. Especially with a time-dependent exposure variable, there is a strong potential for time-dependent confounding, such that a post-baseline confounder may be a common cause of need for surgery and cognitive decline.

In the current study, we hypothesized that dropout due to death or dementia may result in underestimation of the association between surgery with general anesthesia and cognitive decline, as subjects with dementia may have difficulty attending study visits or completing neuropsychological testing and demented subjects likely have lower cognitive scores. While differences in results between analyses assuming MAR and MNAR were small in this study, we have shown that incorporating information about potential dropout mechanisms is an important consideration in clinical research, as differences of this magnitude may have substantial clinical implications in other settings.

## Supplementary material

For supplementary material accompanying this paper visit https://doi.org/10.1017/cts.2020.519. click here to view supplementary material
