# Supplementary material for: Association between surgery with anesthesia and cognitive decline in older adults: Analysis using shared parameter models for informative dropout
Source: J Clin Transl Sci. 2020 Aug 4;5(1):e27. doi: 10.1017/cts.2020.519 (PMC8057447; doi:10.1017/cts.2020.519)
Supplement: Supplementary file 1 [file S2059866120005191sup001.docx]

**Supplemental Material for**

**Association between surgery with anesthesia and cognitive decline in older adults: analysis using shared parameter models for informative dropout**

Katrina L. Devick, PhD^1^, Juraj Sprung, MD, PhD^2^, Michelle Mielke, PhD^3^, Ronald C. Petersen^4^, MD, PhD, Phillip J. Schulte, PhD^1^

^1^Department of Health Sciences Research, Division of Biomedical Statistics and Informatics, ^2^Department of Anesthesiology and Perioperative Medicine, ^3^Department of Health Sciences Research, Division of Epidemiology, ^4^Department of Neurology, Mayo Clinic College of Medicine and Science, Rochester, Minnesota, USA

**Supplemental Material 1:**

Supplemental Table 1: Baseline characteristics according to observed study outcomes

|  | No Observed Death or Dementia | | Death or Dementia | |  |
| --- | --- | --- | --- | --- | --- |
|  | 1-4 Study Visits (N=722) | ≥5 Study Visits (N=784) | Dementia (N=172) | Death (N=270) | Total (N=1948) |
| Age, Median (Q1, Q3) | 80 (75, 83) | 77 (73, 81) | 82 (79, 85) | 82 (78, 86) | 79 (74, 83) |
| Female Sex | 379 (52.5%) | 389 (49.6%) | 81 (47.1%) | 105 (38.9%) | 954 (49.0%) |
| Marital Status |  |  |  |  |  |
| Single | 72 (10.0%) | 76 (9.7%) | 15 (8.7%) | 29 (10.7%) | 192 (9.9%) |
| Married | 445 (61.6%) | 537 (68.5%) | 108 (62.8%) | 143 (53.0%) | 1233 (63.3%) |
| Widowed | 205 (28.4%) | 171 (21.8%) | 49 (28.5%) | 98 (36.3%) | 523 (26.8%) |
| Education Status |  |  |  |  |  |
| Less than 12 years | 82 (11.4%) | 47 (6.0%) | 28 (16.3%) | 34 (12.6%) | 191 (9.8%) |
| 12 years | 286 (39.6%) | 235 (30.0%) | 54 (31.4%) | 87 (32.2%) | 662 (34.0%) |
| 13-15 years | 168 (23.3%) | 201 (25.6%) | 39 (22.7%) | 68 (25.2%) | 476 (24.4%) |
| 16 years and above | 186 (25.8%) | 301 (38.4%) | 51 (29.7%) | 81 (30.0%) | 619 (31.8%) |
| Smoking Status |  |  |  |  |  |
| Never Smoker | 375 (51.9%) | 440 (56.1%) | 86 (50.0%) | 117 (43.3%) | 1018 (52.3%) |
| Former Smoker | 325 (45.0%) | 319 (40.7%) | 84 (48.8%) | 131 (48.5%) | 859 (44.1%) |
| Current Smoker | 22 (3.0%) | 25 (3.2%) | 2 (1.2%) | 22 (8.1%) | 71 (3.6%) |
| Hx Alcohol Problems | 30 (4.2%) | 27 (3.4%) | 5 (2.9%) | 12 (4.4%) | 74 (3.8%) |
| Charlson Index, Median (Q1, Q3) | 3 (2, 5) | 3 (1, 5) | 4 (3, 7) | 5 (3, 7) | 3 (2, 6) |
| Midlife Diabetes | 45 (6.2%) | 35 (4.5%) | 10 (5.8%) | 14 (5.2%) | 104 (5.3%) |
| Midlife Hypertension | 265 (36.7%) | 277 (35.3%) | 55 (32.0%) | 102 (37.8%) | 699 (35.9%) |
| Midlife Dyslipidemia | 294 (40.7%) | 375 (47.8%) | 67 (39.0%) | 108 (40.0%) | 844 (43.3%) |
| Atrial Fibrillation | 119 (16.5%) | 98 (12.5%) | 37 (21.5%) | 78 (28.9%) | 332 (17.0%) |
| Congestive Heart Failure | 81 (11.2%) | 41 (5.2%) | 29 (16.9%) | 69 (25.6%) | 220 (11.3%) |
| Stroke | 47 (6.5%) | 16 (2.0%) | 16 (9.3%) | 33 (12.2%) | 112 (5.7%) |
| Coronary Artery Disease | 281 (38.9%) | 259 (33.0%) | 81 (47.1%) | 155 (57.4%) | 776 (39.8%) |
| *APOE ε4* allele | 206 (28.5%) | 191 (24.4%) | 62 (36.0%) | 59 (21.9%) | 518 (26.6%) |
| Baseline Cognitive Status |  |  |  |  |  |
| Cognitively Unimpaired | 594 (82.3%) | 757 (96.6%) | 68 (39.5%) | 226 (83.7%) | 1645 (84.4%) |
| Mild Cognitive Impairment | 128 (17.7%) | 27 (3.4%) | 104 (60.5%) | 44 (16.3%) | 303 (15.6%) |
| Surgery with Anesthesia in Prior 5 Years | 228 (31.6%) | 234 (29.8%) | 68 (39.5%) | 111 (41.1%) | 641 (32.9%) |
| Surgery with Anesthesia in Prior 10 Years | 363 (50.3%) | 357 (45.5%) | 99 (57.6%) | 160 (59.3%) | 979 (50.3%) |
| Surgery with Anesthesia in Prior 20 Years | 470 (65.1%) | 507 (64.7%) | 131 (76.2%) | 205 (75.9%) | 1313 (67.4%) |

**Supplemental Material 2:**

| Supplemental Table 2: Association of Subject Characteristics and Cognitive Scores | | |
| --- | --- | --- |
|  | *Linear Mixed Effects Model* | *Shared Parameter Model* |
| *Variable* | *est. (95% CI)** | *est. (95% CI)** |
| Intercept-related terms* |  |  |
| Intercept | 4.197 (3.589, 4.805) | 4.173 (3.561, 4.765) |
| Age | -0.058 (-0.066, -0.05) | -0.057 (-0.064, -0.05) |
| Female Sex | 0.15 (0.074, 0.226) | 0.146 (0.071, 0.221) |
| Former Smoker (REF=Non-smoker) | -0.038 (-0.107, 0.031) | -0.042 (-0.108, 0.023) |
| Current Smoker (REF=Non-smoker) | -0.118 (-0.296, 0.06) | -0.111 (-0.28, 0.066) |
| Midlife Diabetes | -0.105 (-0.254, 0.044) | -0.094 (-0.246, 0.047) |
| Midlife Dyslipidemia | 0.004 (-0.067, 0.075) | -0.004 (-0.071, 0.063) |
| Midlife Hypertension | -0.013 (-0.086, 0.06) | -0.008 (-0.075, 0.061) |
| Hx Atrial Fibrillation | 0.058 (-0.036, 0.152) | 0.061 (-0.033, 0.151) |
| Hx Congestive Heart Failure | -0.049 (-0.165, 0.067) | -0.059 (-0.169, 0.061) |
| Hx Stroke | -0.098 (-0.239, 0.043) | -0.096 (-0.238, 0.041) |
| Hx Coronary Artery Disease | -0.047 (-0.12, 0.026) | -0.041 (-0.109, 0.031) |
| Married (REF=Single) | 0.19 (0.074, 0.306) | 0.179 (0.07, 0.29) |
| Widowed (REF=Single) | 0.102 (-0.02, 0.224) | 0.1 (-0.02, 0.216) |
| Hx Alcohol Problems | -0.234 (-0.405, -0.063) | -0.237 (-0.409, -0.06) |
| Education – 12 years (REF= <12 years) | 0.39 (0.27, 0.51) | 0.395 (0.276, 0.52) |
| Education – 13-15 years (REF= <12 years) | 0.598 (0.473, 0.723) | 0.603 (0.479, 0.727) |
| Education – ≥16 years (REF= <12 years) | 0.924 (0.804, 1.044) | 0.935 (0.819, 1.057) |
| Charlson Comorbidity Index | -0.006 (-0.018, 0.006) | -0.004 (-0.016, 0.008) |
| APOE ε4 | -0.13 (-0.203, -0.057) | -0.125 (-0.2, -0.052) |
| MCI | -1.229 (-1.321, -1.137) | -1.216 (-1.307, -1.125) |
| Prior exposure in last 20 years | 0.014 (-0.029, 0.057) | 0.004 (-0.024, 0.035) |
| Slope-related terms** |  |  |
| Years after enrollment | 0.68 (0.564, 0.796) | 0.819 (0.357, 1.307) |
| Years*Age | -0.009 (-0.011, -0.007) | -0.01 (-0.016, -0.005) |
| Years*Female Sex | -0.021 (-0.035, -0.007) | -0.023 (-0.081, 0.038) |
| Years*Former Smoker (REF=Non-smoker) | -0.003 (-0.015, 0.009) | -0.005 (-0.058, 0.05) |
| Years*Current Smoker (REF=Non-smoker) | -0.014 (-0.049, 0.021) | -0.065 (-0.196, 0.068) |
| Years*Midlife Diabetes | -0.023 (-0.05, 0.004) | -0.028 (-0.142, 0.097) |
| Years*Midlife Dyslipidemia | 0.007 (-0.007, 0.021) | 0.023 (-0.033, 0.078) |
| Years*Midlife Hypertension | -0.009 (-0.023, 0.005) | -0.022 (-0.077, 0.033) |
| Years*Hx Atrial Fibrillation | -0.02 (-0.038, -0.002) | -0.018 (-0.093, 0.057) |
| Years*Hx Congestive Heart Failure | 0.009 (-0.015, 0.033) | 0.01 (-0.087, 0.102) |
| Years*Hx Stroke | -0.029 (-0.06, 0.002) | -0.019 (-0.133, 0.093) |
| Years*Hx Coronary Artery Disease | -0.001 (-0.015, 0.013) | -0.017 (-0.074, 0.041) |
| Years*Married (REF=Single) | 0.005 (-0.017, 0.027) | 0.032 (-0.057, 0.127) |
| Years*Widowed (REF=Single) | 0.01 (-0.014, 0.034) | 0.038 (-0.057, 0.137) |
| Years*Hx Alcohol Problems | -0.019 (-0.05, 0.012) | -0.009 (-0.14, 0.124) |
| Years*Education – 12 years (REF= <12 years) | -0.027 (-0.052, -0.002) | -0.039 (-0.134, 0.053) |
| Years*Education – 13-15 years (REF= <12 years) | -0.04 (-0.065, -0.015) | -0.052 (-0.152, 0.048) |
| Years*Education – ≥16 years (REF= <12 years) | -0.034 (-0.058, -0.01) | -0.055 (-0.152, 0.036) |
| Years*Charlson Comorbidity Index | -0.001 (-0.003, 0.001) | -0.002 (-0.011, 0.007) |
| Years*APOE ε4 | -0.033 (-0.047, -0.019) | -0.027 (-0.084, 0.031) |
| Years*MCI | -0.071 (-0.093, -0.049) | -0.107 (-0.179, -0.037) |
| Years*Prior exposure in last 20 years | 0.002 (-0.01, 0.014) | -0.023 (-0.063, 0.017) |
| Change in Slope after Exposure*** |  |  |
| **Years after a Post-enrollment exposure** | **-0.063 (-0.079, -0.047)** | **-0.081 (-0.136, -0.026)** |

*Intercept-related terms are those that relate to the estimated intercept of the linear mixed effects model (expected cognitive z-scores at enrollment). Estimates reflect standard deviations (SDs) associated with the given variable.

**Slope-related terms are those related to the estimated change in cognitive z-scores (SD per year) after enrollment.

***Change in Slope after Exposure is the primary exposure of the current investigation, assessing the association with a post-enrollment exposure to surgery with general anesthesia and subsequent change in cognitive z-scores.

Supplemental Material 3: R Code

library(JMbayes)

#Dataset lng

##one row per observation (multiple rows per subject)

## 'id' is the subject identifier

## zglobal is the primary outcome

## year is time after enrollment for the given study visit

## yearsafter is the time after a post-enrollment exposure

## Other covariates: age, female gender, smoking status, etc.

MixedModelFit<-lme(zglobal ~

age + gender +

factor(smokestat) +

midlifediabetes + midlifehtn + midlifedyslipid +

bsel_afib + bsel_chf + bsel_stroke + bsel_cad +

factor(maritalgrp) + alcoholproblemhx +

factor(educgrp) + bsel_ch_index +

any_e4 +

bsel_mci + pr20_any_anes +

year +

year*age + year*gender +

year*factor(smokestat) +

year*midlifediabetes + year*midlifehtn + year*midlifedyslipid +

year*bsel_afib + year*bsel_chf + year*bsel_stroke + year*bsel_cad +

year*factor(maritalgrp) + year*alcoholproblemhx +

year*factor(educgrp) + year*bsel_ch_index +

year*any_e4 +

year*bsel_mci + year*pr20_any_anes +

yearsafter,

random = ~year+yearsafter|id,

data=lng)

#Dataset srv

##one row per subject

##dthdementia = 1 if death or dementia, =0 if not

##dthdemyrs = years to death or dementia (if dthdementia=1) or censoring

## Other covariates

survFit<-coxph(Surv(dthdemyrs,dthdementia) ~

age + gender +

factor(smokestat) +

midlifediabetes + midlifehtn + midlifedyslipid +

bsel_afib + bsel_chf + bsel_stroke + bsel_cad +

factor(maritalgrp) + alcoholproblemhx +

factor(educgrp) + bsel_ch_index +

any_e4 +

bsel_mci + pr20_any_anes,

data=srv,x=TRUE)

#Fit Shared Parameter Model using JMbayes functions

JMBFit<-jointModelBayes(MixedModelFit, survFit, timeVar = "year",param="shared-RE")

summary(MixedModelFit)

summary(JMBFit)

Supplemental Material 4: Model Formulation

First, we fit linear mixed effects models to assess the relationship between post-enrollment exposure to surgery with general anesthesia and the slope (trajectory) of cognitive z-scores over time. Subject-specific random intercepts, random slopes, and random change in slopes after exposure to anesthesia were included with an unstructured covariance matrix.

Let $i=1,\ldots, N$ denote independent subjects, and $j=1,\ldots, n_{i}$ index the $j$^th^ observation for subject $i$. Define $Y_{ij}$ as the $j$^th^ cognitive z-score for the $i$^th^ subject measured at a study visit occurring at time ${Time}_{ij}$, the time in years from enrollment for the $j$^th^ observation of the $i$^th^ subject.

We define ${TimeAfter}_{ij}$ as a time-dependent variable for the time in years after a post-enrollment exposure to surgery with general anesthesia. That is, ${TimeAfter}_{ij}$ is always zero for a subject who does not have any exposure to surgery with general anesthesia in the study period. For the $i$^th^ subject with exposure to surgery with general anesthesia at time $k$ (in years since enrollment), ${TimeAfter}_{ij}=\max(Time_{ij}-k, 0)$.

Let $\boldsymbol{C}_{i}$ be the vector of adjustment covariates for the $i$^th^ subject at baseline. Then, the linear mixed effects model for the cognitive z-scores assuming missing at random (MAR) is:

|  | $Y_{ij}={(\beta}_{0}+b_{0i})+ {(\beta}_{1}+b_{1i}){Time}_{ij}+\left( \beta_{2i}+b_{2i} \right){TimeAfter}_{ij}+$  $\boldsymbol{\beta}_{\boldsymbol{3}}\boldsymbol{C}_{i}+\boldsymbol{\beta}_{\boldsymbol{4}}\boldsymbol{C}_{i}{Time}_{ij}+\varepsilon_{ij}$ | ^(1)^ |
| --- | --- | --- |

where $\varepsilon_{i}\sim N(0,\sigma^{2}I)$, ${b_{i}=(b}_{0i}, b_{1i}, b_{2i})\sim N(0,D)$ where $I$ is an identity matrix of dimension $n_{i}$ and $D$ is an unstructured covariance matrix. Alternative considerations for the random effects were considered, including exclusion of $b_{2i}$. Akaike Information Criterion (AIC) was used to choose the model fitting the data best.

Further, let $T_{i}$ denote the time to dementia diagnosis, death, or censoring and $\delta_{i}$ be an indicator of dropout (1 for dementia or death, 0 otherwise). Then, the Cox proportional hazards model for the time to (possibly informative) dropout is:

|  | $h_{i}\left( t \right)=h_{0}\left( t \right)exp\left\{ \gamma_{1}b_{0i}+\gamma_{2}b_{1i}+ \gamma_{3}b_{2i}+ \boldsymbol{\gamma}_{\boldsymbol{4}}\boldsymbol{C}_{i} \right\}$ | ^(2)^ |
| --- | --- | --- |

where $h_{i}\left( t \right)$ is the hazard at time $t$ for subject $i$ and $h_{0}\left( t \right)$ is the baseline hazard (i.e. the hazard when all predictors are equal to zero). The linear mixed effects model for cognitive z-scores and the Cox proportional hazards model for time to informative dropout are fit jointly using the *JMbayes R* package. We use the “shared-RE” option for association structure between the two models as described in the formulation of the hazard function (2) above, based on model Deviance Information Criterion (DIC). This shared parameter model is fit with a Bayesian MCMC approach, with diffuse priors. Details can be found in Rizopoulos (2016)^1^.

Operationally, this software package accepts inputs from other R functions nlme::lme() and survival::coxph(). The input models would be of the form (1) for lme() and $h\left( t \right)=h_{0}\left( t \right)exp\left\{ \boldsymbol{\gamma}_{\boldsymbol{4}}\boldsymbol{C} \right\}$ for coxph(). The *JMbayes R* package uses these model formulas and other arguments to fit the joint shared parameter model for (1) and (2).

1. Rizopoulos D. The R Package JMbayes for Fitting Joint Models for Longitudinal and Time-to-Event Data Using MCMC. *Journal of Statistical Software* 2016; **72**(i07).
